# Institutional Defalcations

**Published:** 1914-07-04

**Authors:** 


### Institutional Defalcations.

*To the Editor of* THE HOSPITAL.

SIR,—Now that a point in connection with institutional defalcations has been raised in your columns, may I seek enlightenment from you or your expert readers on a kindred matter? Not many months ago a sub-committee was appointed at a voluntary hospital to inquire into the purchasing and prices of certain stores. This action was taken primarily at the instance of one of the medical staff, who was not satisfied that the hospital was getting value for money. The result of the sub-committee's work and recommendations was that striking economies were effected, and the working expenses of the hospital were very materially reduced. There was very little doubt in the minds of those who went deeply into the matter that one reason for the previous unsatisfactory state of affairs was that the secretary had been taking secret commissions from some of those who supplied the hospital with goods : the latter, in turn, recouping themselves by higher prices against the institution. No proof of this suspicion could be obtained, and the result was that nothing was done in the matter. The official in question was left in unquestioned occupancy of the secretaryship.

The point I would raise is this. Admitting that no charge of corrupt misconduct could properly be brought, or rather sustained, against this officer, would not the management have been justified in giving notice of dismissal on the ground that the interests of the hospital had not been safeguarded as they should have been? In other words, as dishonesty could not be proved, could not incompetence or negligence have been made the reason for a change? And if this is so, were the managers right to overlook the matter and retain the services of such an officer? I put it to your original correspondent, whose case was quite different from this one, of course, that the assistant secretary's responsibility is made much more difficult and onerous if he suspects that defalcations of his superiors may go quite unpunished. In the case I am quoting it is true that it was from another source altogether that suspicions were aroused; but it is obvious that an assistant secretary might be the first to smell a rat if such a case ever arose again, and that his position would be very awkward if as the result of action on his part the scandal was terminated but the culprit remained in office.

I enclose, in confidence, the name of the institution to which I have been referring, and inasmuch as the publication of my name might possibly lead to its identification, I must ask you to refrain from publishing that also.—I am, Sir, yours faithfully,

"TRUSTEE."

[Such an investigation and results as those stated by "Trustee" have often and properly led to changes in the staff.—ED. THE HOSPITAL.]

**[Owing to pressure on our space we have been obliged to hold over the Institutional Library and other regular features.—Ed. The Hospital.]**

